# Predictors of Need for Home Health Services at Discharge for Thoracic Surgery Patients

**DOI:** 10.1016/j.atssr.2024.10.009

**Published:** 2024-10-30

**Authors:** Nataliya Bahatyrevich, Maricruz Diagut, Timothy T. Huynh, Iraklis Erik Tseregounis, Lisa M. Brown, Luis A. Godoy, David T. Cooke

**Affiliations:** 1 Division of General Thoracic Surgery, University of California, Davis, Sacramento, California; 2 Department of Internal Medicine, University of California, Davis, Sacramento, California

## Abstract

**Background:**

In patients undergoing general thoracic surgery, the need for home health (HH) services at discharge increases hospital length of stay. We sought to identify preoperative predictors of HH needs for these patients.

**Methods:**

This was a single-institution, retrospective analysis of patients undergoing elective, nonambulatory thoracic surgical procedures from January 2017 through June 2021. Operations were categorized as “lung,” “esophagus” or “other.” We analyzed and compared preoperative characteristics, intraoperative events, and postoperative complications between HH and No-HH cohorts, including a multivariable logistic regression analysis to identify preoperative predictors for HH need.

**Results:**

We identified 429 patients, with 324 patients (75.5%) discharged without HH and 105 patients (24.5%) discharged with HH. The average length of stay for the No-HH cohort was 3.5 days compared with the HH group of 7.9 days (*P* < .0001). Multivariable analysis revealed age (adjusted odds ratio [aOR], 1.11; 95% CI, 1.10-1.16), clinical cancer stage II (aOR, 6.40; 95% CI, 2.13-19.23), and clinical cancer stage III and higher (aOR, 3.94; 95% CI, 1.12-13.84), preoperative opioid use (aOR, 10.30; 95% CI, 2.24-47.35), higher case mix index (CMI) score (aOR, 1.72; 95% CI, 1.26-2.35), and esophageal operation (aOR, 18.75; 95% C,I 4.70-74.81) as significantly associated with the need for HH.

**Conclusions:**

We identified older age, advanced clinical cancer stage, preoperative opioid use, higher CMI score, and esophageal operation as predictors of the need for HH services. These data may allow for identification of at-risk patients in the ambulatory setting and initiation of HH logistics before admission.

## Visual Abstract

**Figure:**
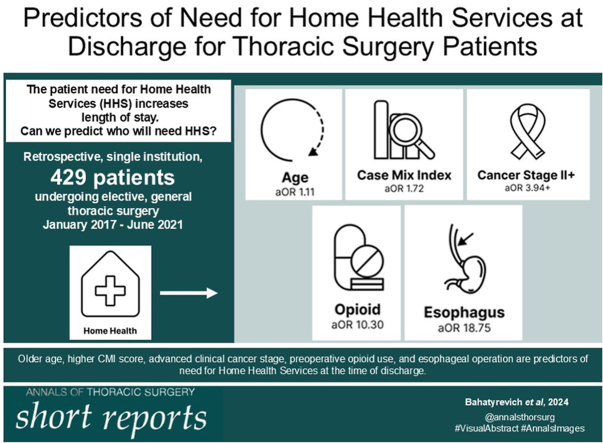

**In Short**

▪ Patients who require HH services have longer postoperative LOS (7.9 days compared to 3.5 days).
▪ Older age, advanced clinical cancer stage, preoperative opioid use, higher CMI score, and esophageal operation are predictors of HH service use at discharge.

The establishment of Enhanced Recovery After Surgery pathways, Integrated Comprehensive Care programs, and other high-value health care models has become increasingly prevalent in managing patients with complex needs, particularly patients undergoing general thoracic surgery. These pathways have shown to be cost-effective and to decrease length of stay (LOS) and hospital readmissions.^1^^,^^2^ A key component of such programs is the integration of home health (HH) services, which typically involve visits to patient’s home several times a week by registered nurses, physical therapists, or occupational therapists. In general thoracic surgery patients, approximately 30% of postoperative patients are reported to require HH services at discharge.^1^^,^^3^

HH use in surgical populations is associated with longer hospital stay.^4^ Furthermore, LOS (25.6% of total hospital cost per case) is a major contributor to hospital costs, as previously shown for video-assisted thoracic surgery lobectomy.^5^ Therefore, decreasing LOS for general thoracic surgery patients improves cost efficiency of care. Anticipating HH needs for thoracic surgery patients in the preoperative ambulatory setting may provide an opportunity for earlier case management planning, thus limiting in-hospital discharge delays. Given limited reports on HH use in general thoracic surgery patients, our objective was to identify independent preoperative predictors of HH disposition for general thoracic surgery patients.

## Patients and Methods

This report describes a retrospective, single-institution cohort study from January 2017 through June 2021 (Institutional Review Board ID 1829810-1, exemption granted June 6, 2022). The inclusion criteria were patients aged 18 years or older who were undergoing elective, nonambulatory general thoracic surgery were discharged either to home or to home with home health (HH) services. Patients discharged with any type of HH services (physical therapy, registered nurse visits, occupational therapy, and others) were included in the HH cohort. We collected the following demographic, intraoperative, and postoperative data by using the electronic medical record system and service line database: age, sex, race, distance from home to the hospital, preoperative comorbidities, and type of operation, broadly categorized as “lung”, “esophagus” and “other,” case mix index (CMI; Vizient, Inc) score, conversion from minimally invasive surgery to open, blood transfusions during operation, and postoperative complications (including but not limited to air leaks, pneumonia, ileus, gastric conduit necrosis, stroke, and arrhythmia). CMI (Vizient, Inc) represents the average diagnosis-related group relative weight for the hospital and is calculated by adding the Medicare Severity-Diagnosis Related Group (MS-DRG) weight for each discharge divided by the number of discharges.^6^ Preoperative comorbidities such as congestive heart failure, coronary artery disease, previous myocardial infarction, and pulmonary hypertension were grouped as cardiac disease. Other preoperative comorbidities, including chronic obstructive pulmonary disease, preoperative home oxygen use, and interstitial fibrosis, were grouped as pulmonary disease. A preoperative history of transient ischemic attack, cerebral vascular accident, myasthenia gravis, dementia, or major psychological disorder were grouped as neurologic disease. Liver disease was defined as any documented preoperative history of abnormal liver function.

Bivariate analysis was used to compare candidate predictors by whether patients received HH services; specifically, *t* tests for continuous predictors (all normally distributed) and Pearson χ^2^ tests for categorical predictors. Predictors with marginal significant differences (P <.2), or clinically relevant ones were included in multivariable analysis. Multiple logistic regression was used to determine independent predictors for HH services. All analyses were conducted using SAS software version 9.4 (SAS Institute). All tests were conducted at the significance level of α = 0.05.

## Results

A total of 429 patients were included in the study; 324 of these patients (75.5%) were discharged without HH services (the no-HH group), and 105 patients (24.5%) were discharged with HH services (the HH group). Of the 429 patients, 283 patients (66%) had a lung operation (categorized as operative area lung), of which 218 (77% of lung operations) were lobectomy and 6 (2% of lung operations) were pneumonectomy, 60 patients (14%) had esophageal surgery (categorized as operative area esophagus), and 3 patients (<1%) had surgery on the chest wall, 59 (14%) were operated on the mediastinum, 22 (5%) had diaphragmatic hernia repair, and 7 (2%) had diaphragm reconstruction (all of these procedures were categorized as operative area other). Of the 60 esophageal surgical procedures, 56 (93% of esophageal operations) were esophagectomy (31 in the HH group, and 25 in the no-HH group); specifically. 26 (46%) had Ivor Lewis esophagectomy, 18 (32%) had transhiatal esophagectomy, 10 (18%) had McKeown (3-hole) esophagectomy, 1 (1.8%) had colonic interposition, and 1 (1.8%) had supercharged jejunal interposition.

Bivariate analysis revealed the HH group to be older (aged 71 ± 8 years vs 62 ± 15 years) and more likely to undergo esophageal operation (32.4% vs 8%) and to have a higher CMI score (3.34 ± 2.33 vs 2.84 ± 0.83) and a higher clinical cancer stage (for stage I, 37.1% vs 47.5%; stage II, 27.6% vs 9.9%; stage III and higher, 18.1% vs 8.3%); they were also more likely to be long-term opioid users before surgery (9.5% vs 1.5%) (Table 1).

**Table 1:** Demographics and Preoperative Bivariate Comparison Between HH and No-HH groups

Variable	Home Health (n = 105)	No Home Health (n = 324)	*P***Value**
Male	54 (51.43)	156 (48.15)	.559
Age, y	71 ± 8	62 ± 15	<.001
White	92 (87.62)	260 (80.25)	.087
Never smoked	27 (25.71)	105 (32.41)	.197
Preoperative opioid use	10 (9.52)	5 (1.54)	<.001
Alcohol use	10 (9.52)	22 (6.79)	.354
Living alone	79 (75.24)	264 (81.48)	.307
Distance to hospital, mi	62.88 ± 53.87	76.79 ± 151.1	.356
ECOG performance status			
0-1	85 (80.95)	304 (93.83)	<.001
2+	19 (18.10)	20 (6.17)	<.001
CMI score	3.34 ± 2.33	2.34 ± 0.83	<.001
Hypertension	71 (67.62)	166 (51.23)	.003
Diabetes	15 (14.29)	64 (19.75)	.209
Cardiac disease	25 (23.81)	44 (13.58)	.013
Pulmonary disease	30 (28.57)	63 (19.44)	.049
Pulmonary embolism or deep vein thrombosis	2 (1.90)	8 (2.47)	.739
Neurologic disease	19 (18.10)	54 (16.67)	.735
Liver disease	1 (0.95)	4 (1.23)	.815
Operative area			
Esophagus	34 (32.38)	26 (8.02)	<.001
Lung	68 (64.76)	215 (66.36)	.382
Other	3 (2.86)	83 (25.62)	<.001
No cancer	18 (17.14)	111 (34.26)	<.001
Cancer stage (clinical)			
1	39 (37.14)	154 (47.53)	.032
2	29 (27.62)	32 (9.88)	<.001
3	19 (18.10)	26 (8.02)	.002
4	0	1 (0.31)	.285

Values are n (%), mean ± SD, or *P*.

HH, home health; CMI, case mix index; ECOG, Eastern Cooperative Oncology Group.

Bivariate comparison of the intraoperative and postoperative characteristics showed that the HH group had longer LOS compared with the no-HH cohort (7.9 ± 6 days vs 3.5 ± 2.5 days) (Table 2). The HH group was also more likely to have an open surgical approach (43.8% vs 22.5%) and unexpected return to the operating room (10.5% vs 1.5%). The HH group was also more likely to have all grouped postoperative complications except for cardiac. The rates of unexpected admission to the intensive care unit and 30-day readmission were similar between the 2 groups.

**Table 2:** Bivariate Comparison of Intraoperative and Postoperative Characteristics

Variable	Home Health (n = 105)	No Home Health (n = 324)	*P* Value
Surgical approach			
Open	46 (43.81)	73 (22.53)	<.001
Minimally invasive	59 (56.19)	251 (77.47)	<.001
ASA class			
2	2 (1.90)	43 (13.27)	<.001
3	96 (91.43)	269 (83.02)	.018
4	7 (6.67)	12 (3.70)	.100
Unexpected return to OR	11 (10.48)	5 (1.54)	<.001
Postoperative complications			
Pulmonary	44 (41.90)	40 (12.35)	<.001
Cardiac	10 (9.52)	17 (5.25)	.117
Gastrointestinal	7 (6.67)	6 (1.85)	.012
Red blood cell transfusion	10 (9.52)	2 (0.62)	<.001
Genitourinary	10 (9.52)	11 (3.40)	.011
Infectious	12 (11.43)	10 (3.09)	<.001
Neurologic	10 (9.52)	12 (3.70)	.019
Unexpected admission to	6 (5.71)	8 (2.47)	.104
30-d readmission	15 (14.29)	19 (5.86)	.014
Intraoperative transfusion	8 (7.62)	1 (0.31)	<.001
Length of stay, d	7.9 ± 6	3.5 ± 2.5	<.001

Values are n (%), mean ± SD, or *P*.

ASA, American Society of Anesthesiologists; ICU, intensive care unit; OR, operating room.

A multivariable analysis of the preoperative factors identified the following as significant predictors of HH services at discharge: age, CMI score, clinical cancer stage II or higher, preoperative long-term opioid use, and esophageal operation (Table 3). Specifically, advanced clinical cancer stage (adjusted odds ratio [aOR], 3.94; 95% CI, 1.12-13.84), esophageal operation (aOR, 18.75; 95% CI, 4.70-74.81), and preoperative opioid use (aOR 10.30; 95% CI, 2.24-47.35) had the highest aORs. Preoperative diabetes mellitus (aOR, 0.41; 95% CI, 0.18-0.95) and neoadjuvant radiation (aOR, 0.12; 95% CI, 0.03-0.52) demonstrated a “protective effect” against HH.

**Table 3:** Multivariable Analysis of Preoperative Variables

Variable	aOR (95% CI)
Sex (male vs female)	0.91 (0.47-1.76)
Ethnicity (White vs non-White)	0.94 (0.37-2.35)
Age	1.11 (1.07-1.16)^a^
Distance traveled	0.996 (0.99-1.00)
Living alone	1.66 (0.81-3.41)
Hypertension	1.06 (0.57-1.99)
Preoperative cardiac disease	1.44 (0.69-3.02)
Preoperative pulmonary disease	1.10 (0.54-2.24)
Preoperative neurologic disease	0.96 (0.43-2.14)
Diabetes	0.41 (0.18-0.95)^a^
Neoadjuvant radiation	0.12 (0.03-0.52)^a^
Clinical cancer stage I vs no cancer	1.46 (0.57-3.74)
Clinical cancer stage II vs no cancer	6.40 (2.13-19.23)^a^
Clinical cancer stage III+ vs no cancer	3.94 (1.12-13.84)^a^
Never smoked	1.43 (0.64-3.18)
Opioid use	10.30 (2.24-47.35)^a^
Alcohol use	1.18 (0.43-3.23)
CMI score	1.72 (1.26-2.35)^a^
ECOG performance score (2+ vs 0-1)	1.87 (0.69-5.10)
Surgical approach (open vs minimally invasive)	1.36 (0.61-3.02)
Esophageal operation	18.75 (4.70-74.81)^a^

aOR, adjusted odds ratio; CMI, case mix index; ECOG, Eastern Cooperative Oncology Group.

a *P* value <.05.

## Comment

Predictors of complex discharge planning have previously been described for nongeneral thoracic patient populations.^4^^,^^7^^,^^8^ However, the reproducibility of these associations has not been investigated in general thoracic surgery patients. Therefore, we specifically analyzed general thoracic surgery patients needing HH services at discharge. We identified that patients with older age, a higher CMI score, clinical cancer stage II or higher, preoperative opioid use, and an esophageal operation were more likely to need HH services at discharge. These predictive factors can be used for disposition planning at the preoperative clinic visit.

Interestingly, patients who had diabetes and received neoadjuvant radiation therapy were less likely to need HH services at discharge. With the increased use of immunotherapy and targeted therapy, it would also be important to investigate in the future the impact of these adjunctive therapies on postoperative recovery of thoracic surgery patients and the need for HH. The protective effect of diabetes on needing HH services is unclear.

### Study Limitations

This study has some limitations, including its retrospective observational design and single institution experience. Some discharge planning decisions and intraoperative practices, such as routine placement of jejunostomy tubes on all patients who undergo esophagectomy, may be institution or region specific and therefore may not be translatable to a broader general thoracic surgery practice.

### Conclusion

Using a multivariable logistic regression model, we identified the following preoperative predictors of HH service use at discharge: older age, advanced clinical cancer stage, preoperative opioid use, higher CMI score, and esophageal operation. Identification of these at-risk patients may aid early disposition planning to expedite insurance preauthorization and HH agency identification and avoid delays in discharge.
